# The present and future of digital health, digital medicine, and digital therapeutics for allergic diseases

**DOI:** 10.1002/clt2.70020

**Published:** 2025-01-03

**Authors:** He Zhang, Yang Cao, Haibo Jiang, Qilin Zhou, Qintai Yang, Lei Cheng

**Affiliations:** ^1^ Department of Otolaryngology‐Head and Neck Surgery The Third Affiliated Hospital of Sun Yat‐sen University Guangzhou China; ^2^ Department of Allergy The Third Affiliated Hospital of Sun Yat‐sen University Guangzhou China; ^3^ Key Laboratory of Airway Inflammatory Disease Research and Innovative Technology Translation Guangzhou China; ^4^ Department of Otorhinolaryngology & Clinical Allergy Center The First Affiliated Hospital Nanjing Medical University Nanjing China; ^5^ China Allergy‐friendly Alliance (CAFA) Nanjing China; ^6^ International Centre for Allergy Research Nanjing Medical University Nanjing China

**Keywords:** allergic disease, chronic disease management, digital health, digital medicine, digital therapeutics

## Abstract

**Background:**

Digital health, digital medicine, and digital therapeutics integrate advanced computer technologies into healthcare, aiming to improve efficiency and patient outcomes. These technologies offer innovative solutions for the management of allergic diseases, which affect a significant proportion of the global population and are increasing in prevalence.

**Body:**

This review examines the current progress and future potential of digital health in allergic disease management. It highlights key advancements, including telehealth, mobile health (mHealth), artificial intelligence, clinical decision support systems (CDSS), and digital biomarkers, with a focus on their relevance to allergic disease management. The role of digital tools in improving treatment adherence, enabling remote care, and integrating environmental and patient data into personalized care models is discussed. Challenges such as data privacy, interoperability, and equitable access are addressed, alongside potential strategies to overcome these barriers.

**Conclusion:**

Digital therapy will play an important role in allergic diseases, and the further development of digital therapies will effectively promote the development of clinical research, digital biomarkers, hypoallergenic environments and digital twins. More research is needed to support the progress of digital therapy for allergic diseases.

## 1 INTRODUCTION

The rapid development of information and communication technology has greatly changed the lifestyles of modern people. The Internet, computers, and smartphones have not only changed daily life but also led to changes in the medical field. How to combine rapidly developing information and computer technology with medicine is a key issue for consideration. This review aims to introduce the basic concepts and development methods related to digital health and focuses on possible applications for patients with allergic diseases.

Digital health is an umbrella term that encompasses electronic health (eHealth) and benefits from areas such as advanced computer sciences (e.g., “big data” and artificial intelligence (AI)). eHealth, as defined by the World Health Organization (WHO),^1^ comprises several components, including eHealth records, telehealth, and mobile health (mHealth).

Digital medicine refers to the use of digital tools to improve the practice of medicine to a high‐definition and far more individualized process. It encompasses the ability to digitize the human body using biosensors that track our complex physiological systems and the means to process the vast amount of data generated via algorithms, cloud computing, and AI. With smartphones as the hub, digital medicine has the potential to democratize medicine, enabling each individual to generate real‐world data and be far more engaged with their health. In addition to new imaging tools, mobile device laboratories, end‐to‐end digital clinical trials, and telemedicine (TM), there is a remarkable array of transformative technology that lays the groundwork for a new form of healthcare.^2^

Digital therapeutics (DTx) are novel therapeutic options that provide treatment for illnesses through software applications (apps) delivered via digital devices. DTx focus on disease treatment and are one of the three core treatment pillars, namely, medical, surgical, and digital therapies.

Digital medicine is a specific means of applying digital health to medical practice, and DTx focus on the diagnosis and treatment of diseases. Allergic diseases affect approximately 40% of the world's population, including most regions around the world, and the incidence rate is increasing annually.^3^ Despite standardized treatment and care guidelines, a significant proportion of patients still have conditions that are not effectively controlled. To this end, new diagnoses, treatments, and nursing strategies to improve the existing situation are needed, and the use of electronic tools may be a promising method.

## 2 TECHNOLOGICAL DEVELOPMENT OF DIGITAL MEDICINE

### 2.1 Telehealth and TM

TM is defined by the Centers for Medicare and Medicaid Services as “the use of telecommunications and information technology to provide access to health assessment, diagnosis, intervention, consultation, supervision and information across distance”. TM is a popular and cost‐effective method for providing patients with access to medical services and providers.

By using telecommunication, TM overcomes distance and time, providing treatment that is as effective as in‐person visits. Virtual TM visits increase the convenience of asthma consultations, allergist practices outside the hospital, and patient management in schools or specific areas.

### 2.2 AI/machine learning (ML)

AI has already exceeded human performance in visual tasks, large‐scale image recognition, and strategy games due to rapid advances in the field of deep learning.^4^ AI‐driven prediction is a rapidly evolving field that has been revolutionized by the advent of deep learning algorithms. While traditional AI models rely on rules provided by human experts, modern AI models can learn from received data and self‐develop complex functions to provide predictions.

For instance, the melanoma detection program based on the input roles of detecting asymmetry and border irregularity is a classic example of a rule‐based AI model. In 2016, Gulshan et al. developed a deep neural network to evaluate images for diabetic retinopathy.^5^ A total of 128,175 images evaluated by 54 board‐certified ophthalmologists and senior ophthalmology residents in the United States were used to train the program. The area under the curve (AUC) of this program for detecting referable diabetic retinopathy reached 0.97–0.99.

### 2.3 mHealth

The term “mHealth” commonly signifies the application of digital health solutions via mobile equipment, where smartphones and wearable devices are the most frequently utilized platforms at present.

#### 2.3.1 Apps

Digital health includes apps, which offer the potential to redesign aspects of healthcare delivery. These apps enable doctors to collect real‐time, longitudinal, and real‐world data. In 2017, more than 318,000 mHealth apps were available for download, nearly double the number available in 2015. For allergic rhinitis (AR) and rhinosinusitis, there are over 1500 apps available in the Google Play and Apple App Store. Moreover, six apps for rhinitis have led to results that are published in the scientific literature: AirRater, AllergyMonitor, AllerSearch, Husteblume, Pollen App, and MASK‐air.^6^

MASK‐air aims to improve the management of AR and asthma multimorbidity via a patient‐centered approach and to facilitate shared decision‐making.^7^ MASK‐air has the following characteristics: (1) it is a free operational app (formerly the Allergy Diary, available for IOS and Android) with more than 50,000 users in 29 countries and is available in 20 languages^8^; (2) it is low cost; (3) it provides quickly available data; (4) it utilizes the visual analog scale (VAS), Control of Allergic Rhinitis and Asthma Test (CARAT), European Quality of Life 5 Dimension (EQ‐5D) questionnaire and Work Productivity and Activity questionnaire for allergic disease monitoring; (5) it provides interoperability with a web‐based physician questionnaire and an electronic clinical decision support system (e‐CDSS) for AR^9^; and (6) it combines air quality and pollen concentration data.

#### 2.3.2 Wearable devices and remote patient monitoring (RPM)

With the development of new technologies such as smart sensing technology, the field of smart wearable devices has developed rapidly in recent years. Wearable devices have played an important role in promoting healthier lifestyles and providing a constant stream of healthcare data. Patient data are collected and sent to doctors for monitoring, chronic disease management, disease diagnosis, and treatment. Smart sensing technology has been applied to some chronic diseases, such as cardiovascular diseases, pulmonary diseases, diabetes, and hypertension. Chronic disease management involves changing passive disease treatment to active health monitoring.^10^

For routine monitoring, noninvasive electrocardiogram (ECG) and Doppler echocardiography are the main means of examining cardiac function.^11^ The 24‐h ambulatory ECG is a relatively advanced wearable medical device used in the clinic. In 2018, the Apple Watch Series 4 was developed, which includes both an ECG app and a watch. Due to advances in sensing technology, inexpensive wearable devices for continuously monitoring heart rate, pulse, oxygen saturation, physical activity, cough sounds, breath sounds, and other characteristics have been developed and can be used for analyzing early lung function deterioration.^12^ It is important to improve the ability of patients with diabetes to self‐monitor and self‐manage their disease by using equipment to measure their blood glucose levels. The traditional way to monitor blood glucose is to directly draw a venous blood sample or prick a finger, while new digital products use indirect measurement methods such as spectrometry, blood substitution (urine, tears, and tissue fluid), counterion electroosmosis and microwave technology.^13^, ^14^

There are already some practices using digital medicine to control cardiovascular disease. For example, the CureApp for hypertension is a therapeutic tool for providing continuous treatment for high blood pressure during both clinic visits and daily life. Non‐pharmacological therapy includes weight loss, the intake of a low‐salt diet, the intake of a low‐fat diet, regular exercise, moderate alcohol consumption, and so on.^15^, ^16^ The CureApp helps patients better implement non‐drug treatments by collecting information on non‐drug factors affecting patients' daily lives, educating patients, and formulating life plans. The efficacy of digital therapy has also been verified in clinical trials. Among 390 patients aged more than 65 years who had essential hypertension but did not receive medical treatment, those assigned to the digital therapy group had a significant decrease in blood pressure after 12 weeks, and the rate of decrease was significantly lower in the digital therapy group than in the control group.

#### 2.3.3 Digital health technology

International asthma guidelines recommend adherence to inhaled corticosteroids (ICSs) and proper inhaler techniques, but poor ICSs adherence has been consistently associated with poor asthma control.^17^, ^18^, ^19^ There are several digital inhaler systems (DISs), RPM devices, and remote therapeutic monitoring (RTM) devices that can enhance ICSs adherence and improve asthma outcomes.^20^ Except of ICSs and short‐acting β2‐agonists, DISs are also equipped with electronic medication monitors, which transmit medication use and inhaler technique data from an asthma app on patients' phones to healthcare professionals in real time. There are a few commercially available digital inhalers in the United States and Europe, such as Capmedic, Houston, and Hailie. Among these, digital platforms for sensors and smartphone apps are available. To determine suitability, several factors must be considered, such as sensor capabilities, inhaler compatibility, the function of the app, and the prescribing and dispensing process.

### 2.4 Clinical decision support system (CDSS)

#### 2.4.1 CDSS

CDSS is a process for enhancing health‐related decisions and actions with pertinent, organized clinical knowledge and patient information to improve health and healthcare delivery, as defined by the Healthcare Information and Management Systems Society (HIMSS). CDSS consists of three parts, including data, algorithms, and reporting.

The data include clinical data, environmental data, and epidemiological data. These data are collected not only from hospitals and health centers but also from digital monitors and the patients themselves. Algorithms are mathematical formulas that use data and generate reports, suggesting diagnostic interpretations or therapeutic decisions.^21^ Algorithms can be optimized based on clinical practice and guidelines. These reporting systems are a way to show results to operators through written reports, suggestions, or therapeutic plans.

Some CDSSs, which aim to collect patient and environmental data, have been used to manage and control AR with drugs or other interventions. The CDSS called @IT‐2020 aims to control seasonal rhinitis and the prescription of allergen immunotherapy. The data include clinical history data, pollen calendar information, allergic sensitization data, component‐resolved diagnostics, and clinical monitoring data. Then, algorithms based on international guidelines, including the Allergic Rhinitis and its Impact on Asthma (ARIA), Global Initiative for Asthma (GINA), and European Academy of Allergy and Clinical Immunology (EAACI) guidelines, report different steps of the diagnostic workup and the connection between the pollen count and the frequency of patients' symptoms.

#### 2.4.2 ML‐enabled clinical decision support

ML models have the potential to determine advanced, complex relationships among enormous numbers of clinical variables, including multimodal data, in ways that traditional statistical risk calculators cannot. ML models have been used in several practices in the field of clinical surgery.^22^ Some mobile apps have been developed using algorithms that can predict surgical outcomes.^23^, ^24^, ^25^ A review of ML models for vascular surgery indicated that some ML models performed better than existing clinical tools, clinicians, and traditional regression models.^26^

## 3 APPLICATION SCENARIO OF DIGITAL HEALTH IN ALLERGY

**FIGURE 1:**
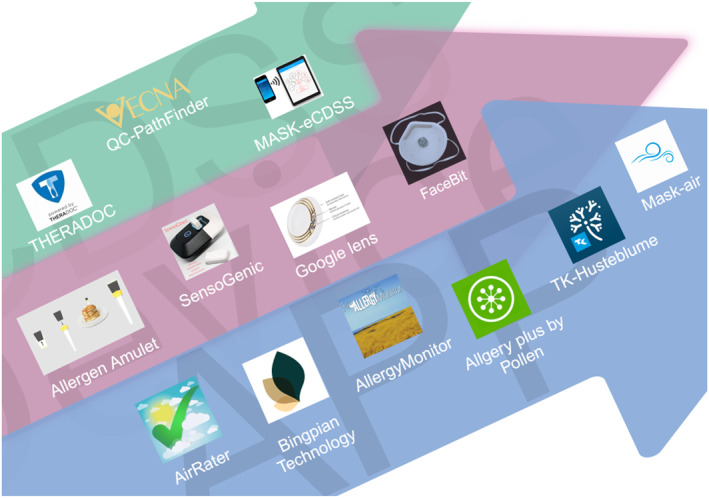
Typical representatives of digital technology applications in the field of allergy and other diseases.

In recent years, digital health has had transformative effects on the management of patients with allergic diseases. TM and emerging technologies are seamlessly integrated into the healthcare system, delivering a diverse range of services and using advanced features specifically tailored to patients with allergic conditions to improve access to healthcare services, ensure the delivery of high‐quality care, and enhance the overall patient experience. A position paper by the American College of Allergy, Asthma and Immunology (ACAAI) showed that TM played an important role in improving the health of patients living in rural or remote areas.^27^ The Task Force of the EAACI also published a position paper describing over 130 allergy‐related mobile apps for allergic diseases.^28^ The relevant apps mentioned above are shown in Figure 1.

### 3.1 Patient‐related service functions

**FIGURE 2:**
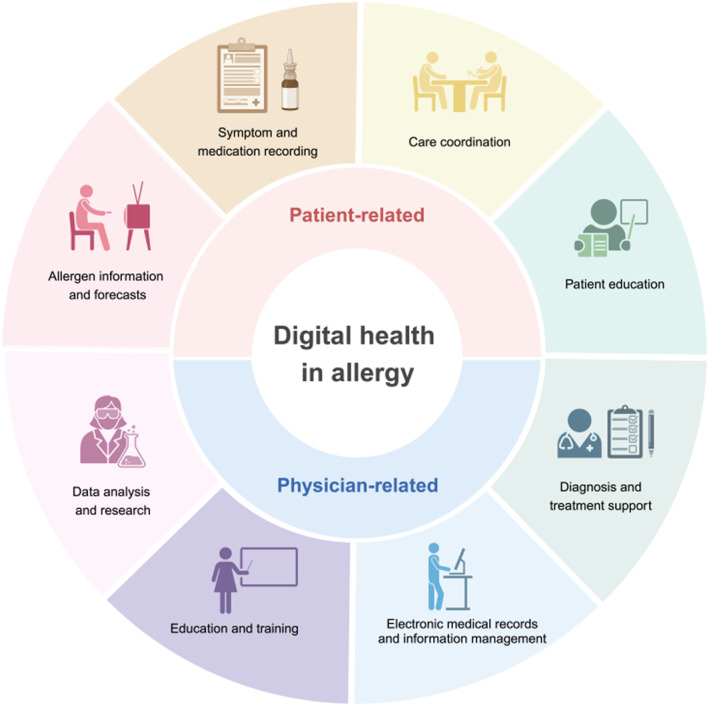
Application scenarios of digital health in allergy for both physicians and patients.

In the field of allergic diseases, high‐quality eHealth tools can contribute to better disease control by providing a deeper understanding of allergic conditions and their triggering factors. These tools can also increase patients' awareness of comorbidities and provide clearer explanations of treatments, thereby improving medication adherence. Furthermore, by establishing personalized follow‐up approaches and providing comprehensive feedback, eHealth tools can help patients achieve better control of their allergic symptoms. Additionally, eHealth applications can serve as a supplement to medical treatment by providing patients with information about non‐pharmacological treatment options, such as lifestyle, diet, and daily exercise, which are also crucial in managing allergic diseases. Therefore, digital healthcare has the potential to significantly improve the overall quality of life of allergy patients, encompassing both physical and psychological well‐being, as well as productivity, including reduced work absences, improved sleep quality, and fewer severe exacerbations. Specific application examples and scenarios are shown in Figure 2 and will be described in the following subsections.

#### 3.1.1 Allergen information and forecasts

##### Pollen

Pollen allergies impact a significant portion of the global population and are expected to increase in prevalence.^28^, ^30^ Therefore, pollen forecasts are highly regarded as essential supplementary services provided to the general public and can play a crucial role in helping individuals with pollen allergies avoid allergens.^31^ Based on user feedback, there are challenges in delivering accurate information and reliable forecasts with traditional methods of aerobiology. This is primarily due to several factors, such as regional variations in allergen content, pollen loads, and pollen allergy symptoms, which can vary from year to year.^32^

Currently, mobile applications such as “Pollen” and “Pollen‐News” have embraced advanced forecasting models that combine real‐time pollen data, historical data for traditional pollen forecasts, meteorological data, and recent symptom data from patients' hay fever diaries. These apps offer a wide range of pollen‐related information, including season countdowns, flowering start dates, various pollen dispersal models, forecast maps, botanical details, and push notification services for patients. Personalized pollen information is accessible through both the pollen information website and a dedicated mobile app.

Glattacker et al.^33^ explored the impact of the Husteblume mobile app on self‐management among patients with pollen allergies. Although they observed no significant changes in quality of life, health literacy, or self‐efficacy before and after the pollen season, over half of the patients (55.9%) reported subjective improvements. Nearly 30% felt they had a better understanding of their condition. While these findings suggest the potential of mobile health apps, the limited number of studies and small sample sizes call for further research to validate these preliminary outcomes.

In a related study, Landesberger et al.^34^ developed the APOLLO app for patients with pollen allergies and asthma. The majority (two‐thirds) of participants felt better informed about their allergies and pollen counts while using the app. However, similar to the Husteblume study, the small sample size underscores the need for larger trials to fully evaluate the app's effectiveness.

Building on these findings, Jones et al.^35^ analyzed over 44,000 symptom reports from the AirRater app to examine the relationship between pollen exposure and respiratory symptoms in Tasmania. Their work demonstrated a clear interaction between pollen levels and PM2.5 pollution, with a non‐linear response that was most pronounced on the day of exposure. These results offer critical insights into how environmental factors amplify allergy symptoms, providing a valuable foundation for developing more sophisticated digital health tools.

These studies, while promising, underscore the need for robust clinical trials to fully assess the efficacy and long‐term benefits of digital health interventions in allergy management. As more patients rely on digital tools, it is vital to ensure that they are supported by evidence‐based practices that improve outcomes and reduce the burden of allergic diseases.

##### Food

Immunoglobulin E (IgE)‐mediated food allergies affect 2%–10% of the population and continue to increase, potentially posing a risk to life safety and quality of life.^36^ Digital technology holds immense potential in assisting patients with the prevention, identification, and management of food allergy reactions. Allergen identification is the category with the highest number of applications. Many of these applications utilize smartphone cameras, allowing users to scan product barcodes to display ingredient lists. The ShopWell ‐ Better Food Choices application allows users to scan barcodes to determine if a product contains specific allergens and discover new allergen‐free alternatives. The Eat! The Gluten‐Free application assists users in finding gluten‐free recipes, products, and services. Importantly, there is currently a lack of applications that can differentiate between food allergies and food intolerances because existing applications are designed for individuals with diagnosed food allergies. Distinguishing between these two conditions could be an important area for future development.^37^

##### Venom

Certain mHealth technologies also show promise in the prevention and management of relatively rare allergies, such as venom allergies. For instance, mobile apps can be utilized to visually document the presence of various Hymenoptera species or uncommon species in specific regions. These data can serve as a foundation for developing warning systems that alert hikers or travelers to the presence of potentially hazardous insects. Furthermore, mHealth has the potential to facilitate the recording and identification of the responsible insect following a sting. Consequently, the implementation of these diverse apps can enhance the identification of patients with venom allergies and contribute to the prevention of severe allergic reactions.^28^

#### 3.1.2 Symptom and medication recording

eHealth records serve as digital repositories for patients' medical histories, diagnoses, treatments, and test results. Authorized healthcare providers can access these records to ensure continuity of care. The AllergyMonitor study aimed to compare different severity scores for AR by examining their correlation with pollen counts at both the population and individual levels. Two groups of children with seasonal AR were monitored using AllergyMonitor, an internet‐based platform. This platform automatically generates symptom scores and symptom‐medication scores based on daily symptom and drug intake data. The results indicated a strong correlation between severity scores and population‐level pollen counts, but individual patients exhibited diverse trajectories. The study concluded that while severity scores yield similar results in large clinical trials, selecting an appropriate scoring system becomes crucial when managing individual patients. The AllergyMonitor platform facilitated the convenient and standardized evaluation of disease severity.^38^

MASK‐air® is a validated mHealth app (classified as a Class IIa medical device) that has been utilized in extensive studies involving more than 58,000 individuals with AR and/or asthma. This app addresses the specific needs of patients with these conditions and is recommended by healthcare experts as a patient‐centered care solution employing digital technology. MASK‐air® has led to groundbreaking discoveries regarding different types of allergic conditions and their management. Analysis of the MASK‐air® data revealed that many AR patients do not adhere to treatment guidelines, often using medication only when experiencing symptoms rather than consistently or as recommended. Moreover, the data demonstrated that medication does not consistently improve symptom control, work productivity, or school performance. A combined symptom‐medication score (ARIA‐EAACI‐CSMS) has been validated for clinical practice and trials. These findings underscore the necessity for changes in the management of AR and asthma.^39^

The Reliever Digihaler System (RDS) offers a comprehensive solution for asthma management, comprising a wireless albuterol inhaler (Digihaler), a smart device application, a Digital Health Platform, and a Dashboard. In a 12‐week study, participants with inadequate asthma control were randomly assigned to use either the RDS or a standard albuterol inhaler. The results demonstrated that RDS users had significantly greater chances of achieving clinically meaningful improvements in asthma control than standard inhaler users after 3 months. These findings emphasize the potential effectiveness of the RDS as a tool for enhancing asthma management and warrant further investigation.^40^

#### 3.1.3 Care coordination

Digital health plays a crucial role in enabling communication and collaboration among healthcare providers, ensuring smooth and coordinated care for patients, particularly those with complex or chronic conditions. Digital health facilitates appointment scheduling, allowing patients to easily book appointments with healthcare providers or specialists. Additionally, it streamlines the registration process for patients upon arrival at healthcare facilities, making it efficient to collect relevant information. Moreover, mHealth technology can be utilized to establish communication with emergency departments or authorities, particularly in isolated regions or situations where immediate assistance is not available during potentially severe allergic reactions. TM and remote monitoring enable virtual healthcare consultations, the remote tracking of vital signs and health data, and the provision of telehealth services, thereby improving access to care, especially for patients in remote or underserved areas. Furthermore, digital health solutions assist in the management of electronic prescriptions, including transmitting prescription information to pharmacies, monitoring medication adherence, and facilitating medication refills.

#### 3.1.4 Patient education

Patient education provides educational materials, resources, and tools to help patients understand their medical conditions, treatment options, and self‐care practices. For example, inhaler use errors are associated with uncontrolled asthma and increased exacerbation rates. According to a systematic review, the use of the correct inhaler technique is still unacceptably low and has not improved over the past 40 years. In a study of asthma patients under specialist care, the correct inhaler technique was not used by 80% of patients. DISs, RPM, and RTM can help patients improve ICSs adherence and inhaler techniques and ultimately improve asthma outcomes and reduce costs. Digital inhalers generally offer a digital platform that includes sensors, a dedicated smartphone software app, and a health care provider dashboard portal, all of which are connected by wireless transmission through a secure cloud server.^40^

### 3.2 Healthcare provider‐related service functions

Healthcare providers might be able to understand disease development more fully considering treatment and the environment and subsequently provide better personalized care. The gathering of and access to a multitude of relevant data will create an opportunity to better monitor the course of disease, comorbidities, adherence, and side effects of therapy over a longer period and enable early identification of patients needing intervention. The use of user‐friendly and simple platforms, together with connectivity to existing healthcare platforms, will also promote the use of time‐saving and time‐effective e‐health tools during outpatient visits. Separate from direct patient care, these quality criteria can facilitate research and registries due to the opportunity to establish larger databases. Big data registries could also benefit from the reporting of disease complications and medication side effects. It's believed that these advantages could lead to increased work satisfaction for healthcare providers.

#### 3.2.1 Diagnosis and treatment support

Digital health tools can offer valuable support to doctors in the decision‐making process regarding diagnosis and treatment. By utilizing medical databases, clinical guidelines, and expert systems, doctors can access real‐time medical knowledge and the latest research findings, enabling them to make more accurate diagnoses and develop more effective treatment plans. A study demonstrated that the use of an electronic diary (eDiary) significantly enhanced the consistency of allergen immunotherapy (AIT) prescriptions among specialists. When allergy specialists combine the ‘traditional approach’ (patient history and skin prick tests) with these diagnostic tools (component‐resolved diagnosis and eDiary), they frequently adjust and standardize their decisions regarding AIT.^41^

According to the 2021 Global Initiative for Asthma guidelines, approximately 17% of adult patients are diagnosed with difficult‐to‐control asthma. This category is defined by the use of high‐intensity treatment (high‐dose ICSs and long‐acting β2‐agonists or medium‐dose ICSs‐long‐acting β2‐agonists plus oral corticosteroids) and poor symptom control. However, importantly, only 3.7% of patients with difficult‐to‐control asthma meet the diagnostic criteria for severe asthma. Severe asthma is characterized by the need for high‐intensity treatment, poor symptom control, good adherence to and the demonstration of the correct inhaler technique. If the patient's condition significantly improves when addressing factors such as adherence to medication and the correct inhaler technique, then the patient's asthma is not classified as severe.^20^

#### 3.2.2 Electronic medical records and information management

Digital health technology enables doctors to manage patients' electronic medical records and healthcare information more easily. Electronic medical records provide quick and accessible patient data, helping doctors better understand patients' medical histories, diagnoses, and treatment records. Additionally, digital information management systems can improve work efficiency and reduce the burden of handling paper documents. Patient feedback and satisfaction surveys can collect input from patients to assess their satisfaction with the healthcare services, identify areas for improvement, and enhance patient‐centered care.

#### 3.2.3 Education and training

Digital health technology provides doctors with opportunities for continuous learning and professional development. Online education platforms, virtual training tools, simulation software, and other digital learning resources can assist doctors in continually updating their knowledge and skills, thereby enhancing their clinical practice.

#### 3.2.4 Data analysis and research

Digital health technology generates a vast amount of medical data that holds great potential for research and analysis. Doctors can use these data to identify patterns in disease occurrence, evaluate the effectiveness of treatments, and conduct clinical research. The utilization of data analysis tools and AI enables doctors to extract valuable insights and information from large datasets. One example is “ResearchKit,” a clinical research support tool that facilitates crucial tasks such as obtaining consent, conducting surveys, and collecting data using smartphones. This simplifies the process of data collection and enables more efficient clinical trials. Furthermore, various apps are being developed and utilized in clinical research on different diseases, such as Parkinson's disease, autism, seizures, and sleep disorders.

In the field of allergic diseases, ongoing research is assessing the usability and impact of a recently developed algorithm for a CDSS for seasonal rhinoconjunctivitis. This algorithm incorporates diagnostic steps such as patient history (anamnesis), skin prick tests or serum IgE tests, component‐resolved diagnosis, and real‐time digital symptom recording (eDiary). To evaluate the algorithm, 46 doctors, including 18 allergy specialists and 28 general practitioners, participated in the study. After receiving educational training on the @IT2020‐CDSS algorithm, the doctors were presented with 10 clinical cases, and they reported their hypothetical decisions regarding AIT prescriptions based on different steps of the algorithm. This study evaluated the usability and perceived impact of the algorithm on doctors' decision‐making processes.

## 4 ADVANTAGES, DISADVANTAGES, AND PROSPECTS OF DIGITAL MEDICINE FOR ALLERGIC DISEASES

### 4.1 Disadvantages and concerns regarding digital therapies for allergic diseases

**FIGURE 3:**
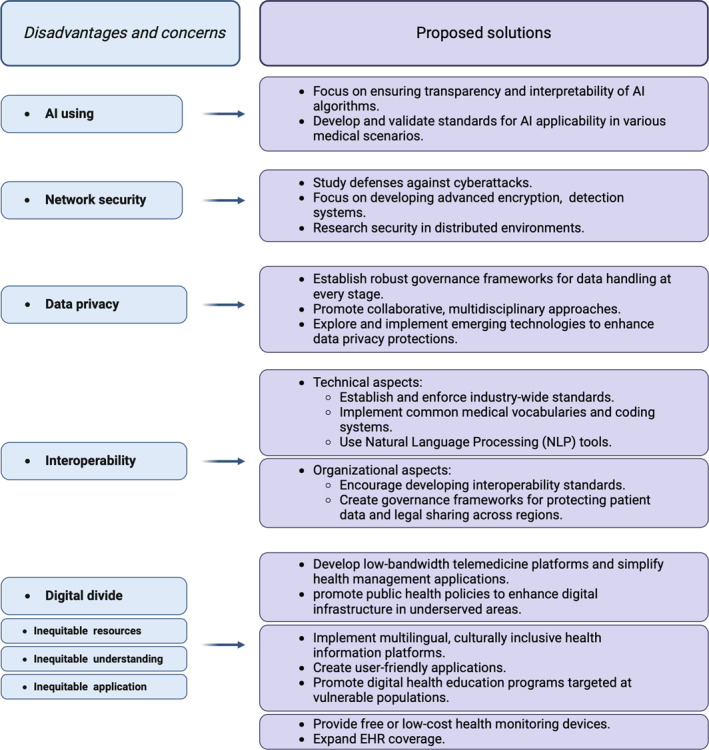
Disadvantages and proposed solutions in digital health, digital medicine, and digital therapeutics. This table outlines the major challenges along with corresponding solutions in AI usage, network security, interoperability, data privacy, and the digital divide, offering practical approaches to address these issues and advance the field.

The following section provides a detailed overview of these disadvantages and their corresponding solutions. These include issues related to AI usage, network security, interoperability, data privacy, and the digital divide, along with practical approaches to tackle each and drive progress in the field. The primary content is summarized in Figure 3, which outlines the main challenges and corresponding solutions discussed in this section.

#### 4.1.1 AI using

AI can update algorithms and iterate itself based on increasing amounts of data to achieve more complete and accurate functions. However, as this process evolves, the use of AI has gradually turned into a black box, and researchers have gradually become less familiar with its understanding and operating principles, which raises concerns. Therefore, it is safer to use AI for repetitive and mechanical tasks. For AI that generates judgments through logic and analysis, more oversight and supervision are still needed.

Research the overuse of AI in healthcare and its potential risks, especially in applications related to diagnosis, decision support, and personalized treatment. The focus should be on the transparency and interpretability of AI algorithms, ensuring that clinicians can understand the recommendations made by AI, thereby avoiding complete dependence on machines. Additionally, we develop and validate standards for the applicability of AI technologies in different medical scenarios, clarifying when AI can assist decision‐making and when human doctors should remain in control.

#### 4.1.2 Network security

When discussing the advantages of digital medicine, we often highlight improvements in adherence, follow‐up, health monitoring, and patient care. However, all these factors require a functioning supportive operational digital infrastructure. Excellent cybersecurity protection is also needed because it is not a question of whether an attack will occur but rather a question of when and where an attack will happen.^42^

Study how healthcare systems can defend against cyberattacks, including ransomware, hacking, and data tampering. The focus should be on developing advanced encryption technologies, blockchain technologies, and AI‐based threat detection and defense systems. Furthermore, as digital therapies expand into TM and virtual health monitoring devices in distributed systems, research the security of these distributed environments, particularly ensuring the safety and integrity of data transmission between patients and healthcare providers.

#### 4.1.3 Data privacy

The advancement of digital health is closely tied to the availability and use of vast amounts of health data. Protecting these data during collection, transmission, storage, and analysis is crucial. Robust governance frameworks must be established at every stage of data handling. However, existing legal and regulatory frameworks for data privacy are still inadequate. Addressing this issue requires a collaborative, multidisciplinary approach involving legislative reform, technological innovation, ethical considerations, and cross‐sector integration.

Efforts to enhance data security have been initiated. For instance, the European Union implemented the General Data Protection Regulation (GDPR) in 2018, which applies to all identifiable personal data. This regulation enforces stricter consent processes, data protection during transfers outside the EU, and individuals' rights to access or erase their data.^43^ In the United States, the Health Insurance Portability and Accountability Act (HIPAA) Privacy Rule serves as foundational legislation for safeguarding health data.^44^ Nonetheless, these regulations are in their early stages and require continuous refinement.

Emerging technologies are also enhancing data privacy protections. Techniques such as differential privacy, federated learning, homomorphic encryption, and swarm learning are being developed to minimize data exposure while still enabling the effective use of sensitive health information in AI systems.^45^, ^46^

#### 4.1.4 Interoperability

Interoperability refers to the ability of different systems to exchange and use information effectively. One major challenge in the widespread implementation of digital medicine is integrating and managing the vast volumes of medical data. This requires establishing standardized communication protocols for data transmission across various devices and institutions. Both syntactic and semantic interoperability are essential for effective data processing and extracting meaningful insights, ensuring that disparate systems can not only share information but also interpret it in a clinically relevant way.

Interoperability also encompasses organizational, legal, and policy aspects. Legally and effectively transferring medical data between organizations, analyzing patient conditions, and achieving comprehensive healthcare coverage necessitate cross‐industry collaboration and regulatory support.^47^

To address the issue of interoperability in digital medicine, both technical and organizational solutions are required to ensure seamless data exchange and utilization across different systems and institutions.

Technical aspects: Establish and enforce industry‐wide standards such as Fast Healthcare Interoperability Resources (FHIR). These protocols provide a framework for exchanging electronic health information and can help achieve syntactic interoperability (the ability to exchange data using the same format and structure). By promoting these standardized protocols, it becomes easier for healthcare providers, payers, and patients to communicate and share data efficiently. Common medical vocabularies and coding systems should be implemented to ensure that exchanged data can be interpreted uniformly across different systems. This ensures that terms used in one system have the same meaning in another, enabling more accurate and clinically relevant interpretations. Natural language processing (NLP) tools can also be used to map disparate terminologies to a common framework, enhancing semantic interoperability.

Organizational aspects: Encourage public‐private partnerships and cross‐industry collaborations to develop and implement interoperability standards. Such initiatives can foster collaboration between technology providers, healthcare organizations, and government entities to establish interoperable systems that benefit all parties. Regulatory bodies should work in tandem with industries to create a governance framework that ensures compliance with these standards while protecting patient data. Develop comprehensive legal frameworks and regulatory policies that govern data sharing and ensure compliance with privacy laws such as HIPAA in the U.S., GDPR in Europe, and other regional standards. These frameworks must specify how data can be shared securely across borders and between different healthcare organizations while ensuring patient consent and confidentiality are maintained.

#### 4.1.5 Digital divide

The digital divide represents the gap between individuals with adequate access to information and communication technology and those with limited or no access.^48^ This issue has gained international recognition as a significant concern for organizations and policymakers.^49^, ^50^ The manifestations of this divide can exacerbate existing societal inequalities, affecting individuals' social and economic capital and their ability to participate in society meaningfully.^51^

Research on the digital divide often highlights sociodemographic and socioeconomic factors such as income, age, education, ethnicity, and urbanization.^52^ In the medical field, the digital divide poses several challenges, each with potential solutions:

1) Inequitable Access to Digital Healthcare Resources: Patients in low‐income or rural areas may lack Internet access or necessary devices, preventing them from utilizing TM or electronic medical record (EMR) systems. Developing low‐bandwidth TM platforms and simplifying health management applications can help bridge this gap. Additionally, public health policies should promote the development of basic digital infrastructure in underserved areas.
2) Inequitable Understanding of Digital Health Information: Effective use of digital health information often requires a certain level of technological knowledge. Some older adults or individuals with lower education levels may struggle to access accurate health information. To address this, multilingual and culturally inclusive health information platforms, user‐friendly applications, and targeted digital health education programs should be implemented.
3) Inequitable Application of Digital Health: Personalized healthcare relies on data and AI algorithms, but if patients cannot accurately collect data or access these technologies, the adoption of personalized medicine may be hindered. Many wearable devices and health monitoring tools depend on digital platforms to transmit data, but economically disadvantaged patients may be unable to afford these devices. Health programs providing free or low‐cost monitoring devices, along with technical support and training, can help reduce this disparity. Expanding electronic health record (EHR) coverage is also crucial to ensure that all social groups can access these technologies.

### 4.2 Potential of digital medicine

#### 4.2.1 Digital clinical trials

Clinical trials are required for causal estimation of the efficacy and safety of new medical treatments, drugs, and devices.^53^ The implementation of traditional clinical trials requires considerable manpower, material resources, and is costly. The identification, recruitment, tracking, and follow‐up of participants demand significant effort, and the time and energy costs for the participants themselves are also substantial. Due to high costs, it is also difficult to conduct large‐scale clinical trials involving medical institutions at all levels. The emergence of digital medicine can solve these problems to a certain extent. Fully digital trials can reach more participants regardless of where they live. Researchers can conduct patient disease monitoring and management assessments more efficiently and in real time and can use various tools for electronic health records, data analysis, and more.

#### 4.2.2 Digital biomarkers

A biomarker is defined as a characteristic that is measured as an indicator of normal biological processes, pathogenic processes, or biological responses to an exposure or intervention, including therapeutic interventions (FDA‐NIH Biomarker Working Group; Biomarkers, EndpointS, and Other Tools (BEST) Resources). Such as blood pressure, blood glucose, IgE, and IL‐4 interventions. Digital biomarkers are defined by the Food and Drug Administration (FDA) as biomarkers collected from digital health technologies.

Several digital biomarkers have been validated. The allergy‐CSMS is a daily, validated, real‐life, digitally enabled, patient‐centered biomarker for any allergic treatment, including AIT,^8^ and it has been used to investigate the effect of AR treatment. A total of 317,176 days of MASK‐air data from 17,780 users in 25 countries were assessed to determine the data‐driven CSMS, and better performance was observed for cluster analysis‐based CSMSs.^54^ The daily electronic asthma control score (e‐DASTHMA) is used to evaluate reported symptoms and medication use in patients with asthma. MASK‐air data for 135,635 days were obtained from 1662 users.^8^

Digital biomarkers have the potential to improve diagnosis, continually monitor patient health, accurately predict outcomes, and rapidly assess exacerbations.^55^ For patients with occupational asthma or allergy multimorbidity, the relative role of nose, lung, ocular, and bronchial symptoms may be complex and overlapping; however, digital biomarkers can combine different symptoms with exposure as well as the control of allergies and asthma. These biomarkers have also been validated as easy and simple tools for these patients.

#### 4.2.3 Creation of hypoallergenic environments and allergen exposure alerts

Allergens are the cause of allergic diseases, and avoiding allergens is one of the most important measures for treating allergic diseases. Applying digital technology to allergen avoidance and creating low‐allergen environments is one of the future development trends. There are already some apps and research that mainly target pollen. More research and apps are needed for important allergens such as dust mites, fungi, and food. Allergen concentrations should be detected and evaluated, and early warning functions should be implemented with the help of algorithms to prevent and treat allergic diseases in patients.

#### 4.2.4 Constructing a personal allergy case—Digital twins

Traditionally, clinical data are collected during visits only once or within a single period. Digital medicine allows for the continuous remote monitoring of patient health data daily, including disease physiology and outcomes. This new era of digital medicine‐generated data can enable digital phenotyping and the quantification of individual patients using multimodal data, and can help build digital twins for precision medicine.^56^

Digital twins include clinical data, daily behavior monitoring, the provision of medication information, follow‐up, health education, follow‐up reminders, etc. For patients with allergic diseases, additional consideration is needed to determine treatment, namely, desensitization treatment and environmental control. Desensitization treatment includes the implementation and efficacy of AIT, while environmental control needs to include allergen monitoring and evaluation of the living environment. All information related to patients and diseases is included in electronic medical records and archived to form the basic framework of digital twins. On this basis, AI or other technologies are combined to realize health risk prediction, provide early disease warning, and other functions, and real‐time patient information is efficiently combined with online or offline medical services, forming a complete closed loop that combines prevention and control.

## 5 EXPLORATION OF DIGITAL HEALTH IN THE FIELD OF ALLERGY IN CHINA

According to World Allergy Organization, about 22% of people suffer from IgE‐mediated allergic diseases, which means that about 310 million people in China are affected by allergic diseases.^3^ However, there are only 90 allergy departments and 350 allergists in China, and the doctor‐patient ratio is estimated to be 1:4,000,000.^57^ The lack of allergy specialists calls for extra aid of digital medicine in patient education and disease monitoring.

In recent years, efforts in China have focused on improving pollen monitoring accuracy and using DTx for AR management. The Beijing Tongren Hospital research group has established AR prevalence related to meteorological data in northern China and developed the “Beijing Pollen Health App” jointly with Beijing Meteorological Service.^58^ In 2021, Lei and colleagues conducted a comparative analysis of the effects of fine particulate matter (PM2.5) and coarse particulate matter (PM2.5‐10) exposure on lung function in adult asthma patients.^59^ This study was based on a dynamic lung function monitoring database of 4992 adult asthma patients from 25 cities in China, combined with atmospheric pollution data. This research contributes to guiding asthma patients in taking targeted protective measures to prevent acute asthma attacks caused by environmental factors. Additionally, Ma and colleagues conducted a preliminary study on the use of portable spirometers in children with asthma for self‐monitoring of ventilatory function.^60^ Measuring respiratory function at home can monitor the severity of asthma attacks and assist patients in self‐managing the disease.

Currently, the development of digital medicine in the field of allergology in China lags behind that of developed countries in Europe and North America. This gap is primarily evident in technological research and innovation, data sharing and privacy protection, market maturity, and integration into the healthcare system. However, it also presents immense potential, which can be attributed to China's vast market size, government policy support, rapid technological innovation, and the gradual improvement of the industrial chain. Coupled with advanced experience gained through international cooperation, China is expected to quickly narrow the gap in the field of digital allergology and demonstrate unique strengths and development prospects. With the acceleration of localized innovation, China's future development in this field should not be underestimated.

## 6 CONCLUSION AND FUTURE PERSPECTIVES

This review introduced various types of DTx and their applications for a range of diseases and discussed their advantages and disadvantages. DTx will play an important role in allergic diseases, and the further development of it will effectively promote the development of clinical research, digital biomarkers, allergy‐friendly environments and digital twins. More research is needed to support the progress of DTx for allergic diseases.

## AUTHOR CONTRIBUTIONS

HZ and YC contributed to the writing of this article and are co‐first authors. HBJ and QLZ participated in the revision of the article. QTY and LC led the entire study and conducted rigorous review and editing of the manuscript; they are co‐corresponding authors. All authors have read and agreed to the final version of the manuscript.

## CONFLICT OF INTEREST STATEMENT

The authors declare no conflicts of interest.

## Data Availability

Data sharing is not applicable to this article as no datasets were generated or analyzed during the current study.
